# Validation of Recombinase Polymerase Amplification with In-House Lateral Flow Assay for *mcr-1* Gene Detection of Colistin Resistant *Escherichia coli* Isolates

**DOI:** 10.3390/antibiotics13100984

**Published:** 2024-10-17

**Authors:** Naeem Ullah, Nutchaba Suchanta, Umaporn Pimpitak, Pitak Santanirand, Nutthee Am-In, Nuntaree Chaichanawongsaroj

**Affiliations:** 1 Center of Excellence for Innovative Diagnosis of Antimicrobial Resistance, Department of Transfusion Medicine and Clinical Microbiology, Faculty of Allied Health Sciences, Chulalongkorn University, Bangkok 10330, Thailand; naeem.bch@gmail.com (N.U.); nutchaba.suchanta@hotmail.com (N.S.); 2 Institute of Biotechnology and Genetic Engineering, Chulalongkorn University, Bangkok 10330, Thailand; umaporn.p@chula.ac.th; 3 Department of Pathology, Faculty of Medicine Ramathibodi Hospital, Mahidol University, Bangkok 10400, Thailand; pitak.san@mahidol.ac.th; 4 Department of Obstetrics Gynaecology and Reproduction, Faculty of Veterinary Science, Chulalongkorn University, Bangkok 10330, Thailand; nutthee.a@chula.ac.th

**Keywords:** *Escherichia coli*, recombinase polymerase amplification, lateral flow assay, mobile colistin resistance 1 (*mcr-1*) gene, point-of-care testing

## Abstract

Background/Objectives: The emergence of the mobilized colistin resistance 1 (*mcr-1*) gene, which causes colistin resistance, is a serious concern in animal husbandry, particularly in pigs. Although antibiotic regulations in many countries have prohibited the use of colistin in livestock, the persistence and dissemination of this plasmid-mediated gene require effective and rapid monitoring. Therefore, a rapid, sensitive, and specific method combining recombinase polymerase amplification (RPA) with an in-house lateral flow assay (LFA) for the *mcr-1* gene detection was developed. Methods: The colistin agar test and broth microdilution were employed to screen 152 *E. coli* isolates from pig fecal samples of five antibiotic-used farms. The established RPA-in-house LFA was validated with PCR for *mcr-1* gene detection. Results: The RPA-in-house LFA was completed within 35 min (20 min of amplification and 5–15 min on LFA detection) at 37 °C. The sensitivity, specificity, and accuracy were entirely 100% in concordance with PCR results. No cross-reactivity was detected with seven common pathogenic bacteria or other *mcr* gene variants. Conclusions: Therefore, the in-house RPA-LFA serves as a point-of-care testing tool that is rapid, simple, and portable, facilitating effective surveillance of colistin resistance in both veterinary and clinical settings, thereby enhancing health outcomes.

## 1. Introduction

Colistin has been widely used in veterinary medicine for prophylactic and therapeutic purposes, especially in pig production [1]. This last-resort antibiotic is critical for treating severe infections caused by multidrug-resistant gram-negative bacteria. However, its clinical usage is limited due to its high toxicity [2]. The increasing global prevalence of colistin resistance has emerged as a critical concern in the One Health perspective [3]. Particularly, horizontal transfer rapidly disseminated plasmid-mediated mobile colistin resistance (*MCR*) genes across all sectors, including animals, foods, farms, humans, and the environment [2].

The *mcr* genes encode phosphoethanolamine transferase enzymes that add a phosphoethanolamine (PEtN) to lipid A of lipopolysaccharide (LPS) in the outer membrane of gram-negative bacteria. This neutralizes the negative charge on the bacterial surface, disrupting the electrostatic binding between the cationic peptide of colistin and LPS [4]. To date, 10 *mcr* variants (*mcr-1* to *mcr*-*10*) have been found, which contained 81%, 34%, 33%, 31%, 82%, 29%, 31%, 36%, and 29% identical amino acid sequences to *mcr-1*, respectively. The global prevalence of overall *mcr* genes is 4.7% with different distributions of 3.6% of *mcr-1* and 1.1% of *mcr-9* in Enterobacterales, 4.5% of *mcr-3* in *Aeromonas* spp., and 0.2% of *mcr-10* in *Enterobacter kobei* [4]. The prevalence rates of *mcr*-positive organisms from the poultry sector in low- and middle-income countries during the last half-century ranged from 0.51 to 58.8% [5]. The *mcr-1* gene, which was first discovered in *Escherichia coli* isolated from a Chinese pig, is the most predominant and rapidly spreading globally in animals and environments. In addition, the *mcr-1* distribution in the food chain is a serious alarm for rapid and effective epidemiological control [6].

In 2021, two phenotypic methods including broth disk elution and agar test for colistin resistance were approved by the Clinical Laboratory Standard Institute (CLSI) [7]. Disk diffusion is not recommended for colistin susceptibility testing due to poor drug diffusion in agar, which is prone to high error rates of false susceptibility [8]. Broth microdilution (BMD) remains a reference method considered appropriate for analyzing colistin susceptibility, according to joint CLSI and European Committee on Antimicrobial Susceptibility Testing [9]. However, phenotypic detection was labor-intensive, time-consuming, and had a low detection limit for low resistance or heteroresistance. The advantages of several genotypic detections are attractive due to their rapid, high sensitivity and specificity, as well as the ability to identify novel resistance mechanisms. Although PCR-based methods are well established for *mcr* gene detection, the assays require specialized and expensive equipment. For example, the duplex real-time PCR assay yielded 98% and 96% amplification efficiency for *mcr-1* and *mcr-2* genes, respectively, with 100% sensitivity and specificity [10]. Loop-mediated isothermal amplification (LAMP) and recombinase polymerase amplification (RPA) are rapid, equipment-free isothermal amplification techniques that have been developed for *mcr-1* gene detection in clinical and environmental samples. The LAMP reaction for detection of the *mcr-1* gene showed a detection limit of 0.2 pg/µL, which was 10-fold greater than PCR [11]. Recently, one-tube RPA-CRISPR-Cas12b-based detection systems enabled *mcr-1* and *tet*(X4) detection with a sensitivity of 6.25 and 9 copies, respectively. The spiked pork samples with the *mcr-1*- positive strain were lowest detected at 7 CFU/g [12].

The lateral flow assay (LFA) is a simple, rapid, cheap, and portable tool for point-of-care testing (POCT) or field studies. The immunological principles based on antigen-antibodies or probe-target DNA hybridization are utilized to visually detect specific analytes in the samples [13,14]. The combination of RPA and LFA is a powerful tool for nucleic acid detection, with various applications including pathogen detection, food safety testing, environmental monitoring, etc. [15]. Recently, multiple antibiotic resistance genes (*mcr-1*, *bla*_NDM-1_, and *tet(X4)*) were simultaneously detected by RPA-LFA [16]. The multiplex RPA with commercial LFA was developed to detect the *bla*_CTX-M_, *bla*_SHV_, and *bla*_OXA_ genes from *Escherichia coli* isolated from pork meat and fecal samples. The RPA amplicons were rapidly amplified within 30 min in a heat-dried bath with 99.2% sensitivity and 100% specificity [17].

This study aimed to develop RPA combined with in-house lateral flow assay (RPA-LFA) for detecting the *mcr*-*1* gene in commensal *E. coli* isolated from pig farms using antibiotics. The developed RPA-LFA was validated with broth microdilution and PCR techniques. Our RPA-in-house LFA was established first, which serves as a potentially promising rapid screening for the presence of the *mcr-1* gene. This assay contributes to improved antibiotic stewardship and effective monitoring of colistin resistance, especially in low-resource settings.

## 2. Results

### 2.1. Phenotypic Detection of Colistin-Resistant E. coli Isolated from Antibiotics Using Pig Farms

**Figure 1:**
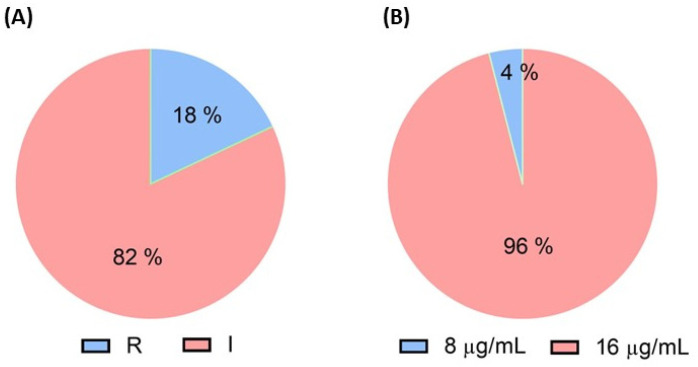
Phenotypic and genotypic resistance analysis in *E. coli* isolates from pigs, analysis by (**A**) colistin agar test, and (**B**) broth microdilution method.

Out of 152 isolates, only 28 (18%) from a single farm exhibited resistance at minimum inhibitory concentration (MIC) ≥ 4 µg/mL (Figure 1A). Among these 28 isolates, 27 (96%) had a MIC of 16 µg/mL, and 1 (4%) had a MIC of 8 µg/mL (Figure 1B).

### 2.2. Optimization of Key Parameters in In-House LFA Development

**Figure 2:**
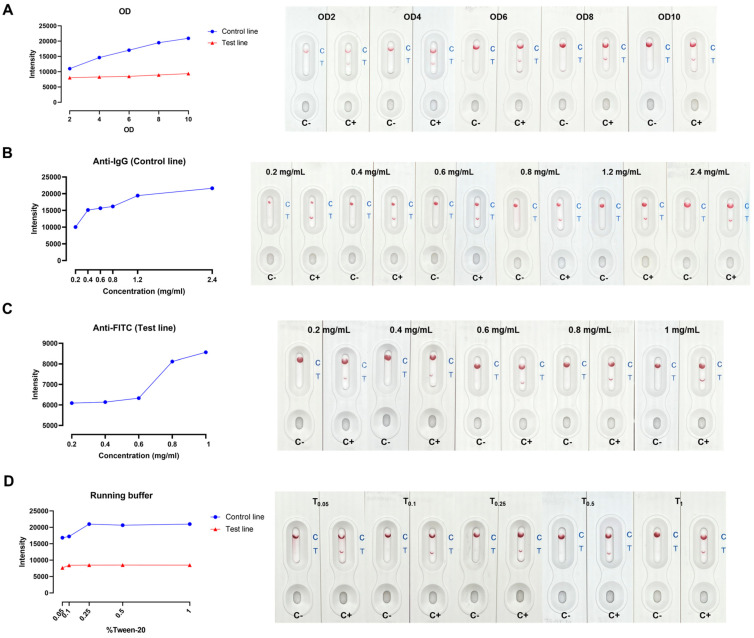
In-house LFA optimization (**A**) anti−biotin conjugated AuNPs concentrations, (**B**) anti−IgG antibody concentrations, (**C**) anti−FITC concentrations, and (**D**) % tween−20 in running buffer.

It is crucial to optimize several parameters in LFA development to obtain a great signal-to-noise ratio and capillary flow rate. The key factors, including anti-biotin conjugated gold nanoparticle (AuNP) concentration embedded in the conjugated pad, the antibodies concentrations at test and control lines, and (0.05–1% *v*/*v*) tween-20 in running buffer, were addressed. The intensity of both test and control spots was maximized when using anti-biotin conjugated AuNP at OD 10 (Figure 2A). The intensity of control spots correlated with the anti-IgG antibody concentrations from 0.2 to 2.4 mg/mL (Figure 2B). Fluorescein Isothiocyanate (FITC) is a fluorescent dye frequently employed to label proteins, antibodies, and other molecules in biological studies to visualize and monitor biological processes. Although the anti-FITC at 1.0 mg/mL showed the maximum intensity, the distinct red spots could be observed by the naked eye at 0.6–0.8 mg/mL anti-FITC (Figure 2C). At 0.1% tween-20, the background was clear, and the result was visible through visual detection. The intensity of test spots did not differ between 0.1–1% tween-20 (Figure 2D).

### 2.3. Optimization of mcr-1 Primer Concentrations and Conditions in RPA-in-House LFA

**Figure 3:**
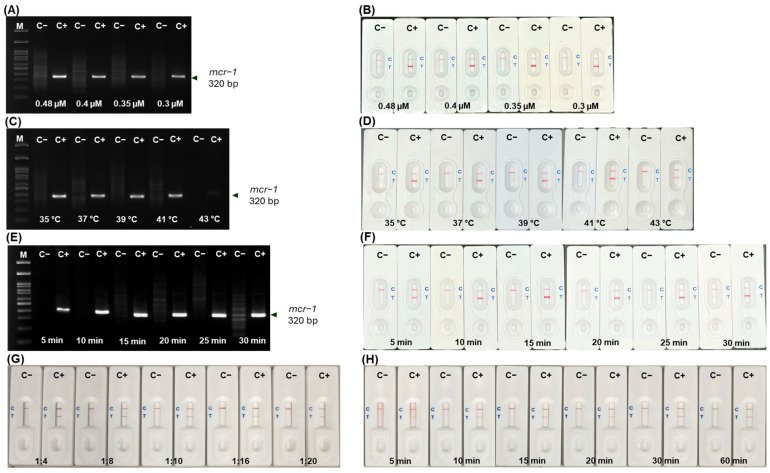
Optimization of RPA and LFA for *mcr*-*1* detection on AGE and LFA, respectively. Primer concentrations (**A**,**B**); temperature (**C**,**D**); and incubation time (**E**,**F**); RPA product: buffer ratio at 1:4–1:20 (**G**) and detection time on LFA (**H**). [M, 100 bp Marker; C−, no template control; C+, positive DNA control].

The optimum concentration of *mcr*-*1* primers required in the RPA reaction was 0.3 µM. When the primer concentration was higher than 0.3 µM, it made non-specific bands on agarose gel electrophoresis (AGE) (Figure 3A) and false positive results on LFA in the no template control (Figure 3B). The *mcr*-*1* product could be successfully amplified at temperatures ranging from 35 °C–43 °C. However, a very faint band on AGE was observed at 43 °C (Figure 3C), while an apparent result on LFA was detected (Figure 3D). The product was amplified within 5–30 min; however, an incubation time >25 min led to a non-specific band on AGE (Figure 3E) and false positive results on LFA (Figure 3F).

The ratio of RPA product to running buffer at 1:4–1:20 showed distinct LFA results between negative and positive samples (Figure 3G). A ratio of 1:10 could detect the *mcr*-*1* gene within 10–60 min with no false positive or negative results and a clear background (Figure 3H). Finally, the optimized RPA conditions were set at 37 °C for 20 min using 25 ng/µL template DNA, a 1:10 ratio of RPA product to running buffer to LFA was applied, and the results were interpreted within 10–15 min.

### 2.4. Limit of Detection (LOD) and Cross-Reactivity of RPA-LFA

**Figure 4:**
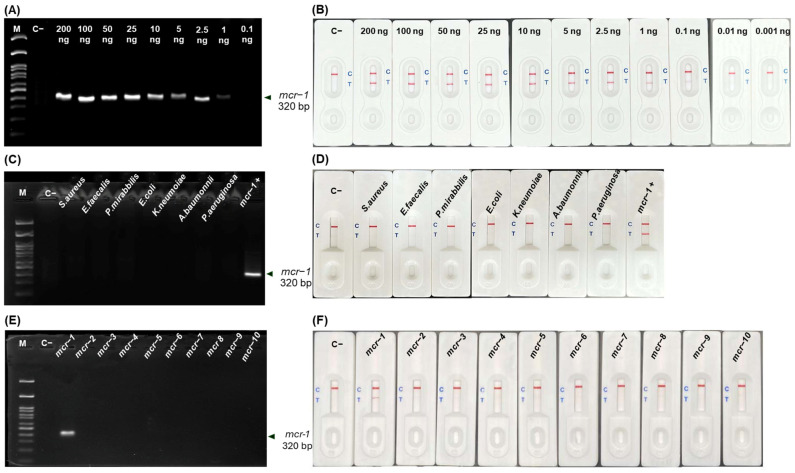
LOD and cross-reactivity for *mcr*-*1* detection by RPA-LFA. AGE results at different DNA template concentrations (0.1–200 ng) (**A**) and corresponding LFA (**B**). AGE results with 7 bacterial strains (**C**), *mcr-1* to *mcr-10* (**E**) and corresponded with LFA (**D**,**F**) detection. [M, 100 bp Marker; C−, no template control; C+, positive].

The limit of detection (LOD) for *mcr*-*1* was 1 ng on AGE (Figure 4A), and 0.1 ng on LFA (Figure 4B), respectively, underlying the superior sensitivity of LFA.

All tested standard strains showed no cross-reaction (Figure 4C,D). Moreover, only *mcr*-*1* was detected by both AGE (Figure 4E) and LFA (Figure 4F), with no detection of other *mcr* types (*mcr*-*2* to *mcr*-*10*). Thus, our developed in-house LFA is specific only for *mcr*-*1* detection.

### 2.5. Detection of mcr-1 Gene from Pig and Clinical Samples by RPA-LFA

Table 1 shows that all 90 samples (28 pig samples and 62 clinical isolates) demonstrated colistin resistance, while the phenotypic BMD method found 33 pig samples to be colistin susceptible. Based on the phenotypic BMD method, our RPA-in-house LFA had a sensitivity of 72.2% [CI 61.78 to 81.15%] and a specificity of 100% [CI 89.42 to 100%]. This was because 28% (*n* = 25/90) of the cases of colistin resistance were not *mcr-1*. A comparison between PCR results for all 123 samples and our RPA-in-house LFA results revealed 100% sensitivity, specificity, and accuracy for *mcr-1* detection.

## 3. Discussion

The emergence of the *mcr-1* gene and its variants on a global scale raises serious concerns about losing colistin, a last-resort antibiotic for the treatment of MDR-gram-negative bacteria [1]. Although many countries, such as China, Thailand, Japan, Vietnam, Indonesia, Malaysia, India, and Australia, have banned the use of colistin in feed as a growth promoter in livestock since 2017, the dissemination and persistence of the *mcr* gene family in one health system are still emerging [18]. The rapid, affordable, and simple genotypic approaches for *mcr* gene detection are deemed crucial for effective colistin resistance control and epidemiological investigations.

Following the withdrawal of colistin in 2017, the longitudinal monitoring of *mcr*-positive *E. coli* isolates in central Thailand pig farms revealed a fluctuating incidence rate of *mcr* genes in pigs, ranging from 3.33% to 53.3% between 2017 and 2020. Similarly, the variation rates of *mcr* genes in China during 1993–2019, were suggested to be due to antibiotic policies, host origin, temporal trends, and geographical distribution [19]. Interestingly, in 2020, colistin-resistant *E. coli* isolates were observed in 33.3% of pigs and 60% of wastewater samples, with all carrying the *mcr-1* gene [20]. Following the colistin usage ban in China, the production and sale of colistin sulfate significantly decreased, resulting in a significant decline in the *mcr-1* gene from 34.0% in 2015–2016 to 5.1% in 2017–2018 [21]. Although colistin-resistant *E. coli* was identified in only one out of five farms in our study, the prevalence of *mcr-1* in our pig farms was 18%, with 96% of them exhibiting a MIC of 16 µg/mL which is two folds higher than the breakpoint value. The *mcr* genes were present in merely 11.3% of fecal samples from pigs collected from 74 Spanish industrial farms during 2020–2022. Of all, 7.5% of the isolates had MIC at 8 µg/mL [22]. The high-level colistin-resistant strains in our study indicate a high threat level and long-term antibiotic use in pig farms which should be seriously monitored in clinical implications.

Due to the high prevalence rate of the *mcr-1* gene in *E. coli* isolates from farms, animals, and environments worldwide, particularly in pig production, we focused our RPA-in-house LFA method on its detection [23,24]. This plasmid-borne *mcr* gene can be horizontally transferred between bacteria and can rapidly disseminate. Several nucleic acid amplification methods have been established for *mcr* gene detection with different efficiency. PCR and real-time PCR, while effective, are not ideal for field applications due to their reliance on specialized instruments and lower efficiency compared to loop-mediated isothermal amplification (LAMP) and RPA-based isothermal amplification methods [11,25,26]. The LAMP techniques are known for their high sensitivity and specificity; however, the reaction typically involves the use of 4–6 complex primers and longer amplification times compared to RPA [25]. The commercial RPA-LFA (TwistAmp^®^nfo kit, Cambridge, UK) and other LFA platforms have been investigated for diverse microorganism detection. The RPA-LFA assay is typically conducted at 37 °C with an amplification time of 15–30 min and visual inspection within 5–15 min. Various parameters affect RPA assay efficiency and analytical sensitivity including primer composition and length, temperature, agitation, incubation time, sample types, and presence of inhibitors. Generally, the optimum length of RPA primer should be 30–35 bp, which is suitable for the formation of stable nucleoprotein filament. However, several reports demonstrated that normal PCR primers can be efficiently used as well. A GC content below 30% or above 70% is not recommended. The optimum length of amplicon is between 80–400 bps, especially 100–200 bps. Generally, the operating temperatures fall between 37 °C and 42 °C, allowing for the use of even body heat without the need for precise temperature control. However, the reaction was unstable if the ambient temperature fell below 30 °C. Agitation is another crucial factor that increases interaction and helps overcome the viscosity of RPA components. The TwistDx^TM^ recommended two mixing steps at the beginning of the process and 4–5 min after initiation. In addition, constant shaking throughout the RPA reaction enhances the reaction rate, produces more stable signals on LFA, and improves sensitivity. RPA amplicons can be generated in 3–4 min, and ATP hydrolysis completes within 25 min. However, this reaction time depends exclusively on the number of initial copies of genetic materials and the size of the amplicons [27].

LFA is an end-point detection method that is superior to AGE, which requires a product purification step to avoid smeared bands on the gel due to the presence of proteins and crowing agents [27]. Moreover, the lower LOD and sensitivity of AGE detection derive from product loss during the purification step and depend on the gel staining dye. The RPA amplification and visual detection on LFA can be achieved in less than 1 h with high sensitivity and specificity. An antibody labeled with antigen-specific gold nanoparticles is a common LFA platform. Specific antibodies label two markers at both 5′ ends of RPA products, ready for detection in a sandwich format at the test and control lines. False positive results on LFA can occur due to primer-dimer formation, particularly at high primer concentrations. It is recommended to dilute the RPA amplicon with the running buffer before applying it to the strip to improve its wicking performance and avoid faint ghost band effects [27]. The ratio of the RPA product and running buffer must be optimized to obtain the maximum sensitivity and specificity with the lowest detection time. Our RPA-in-house LFA determined the LOD of *mcr-1* to be 0.1 ng/25 µL, with 100% sensitivity and specificity when validated with PCR-AGE results of 123 *E. coli* isolates. Recently established RPA-based methods, including RPA-CRISP/Cas12a [28], RPA combined with a horseradish peroxidase (HRP)-catalyzed lateral flow immunoassay (LFIA) biosensor [29], and the utilization of a TwistAmp nfo kit combined with an LFD test strip [16], have all shown promising effectiveness for *mcr-1* detection. The RPA combined with a lateral flow biosensor was validated with standard PCR assay for the detection of extensively drug-resistant genes in 95 samples of *Enterobacteriaceae*. The sensitivity and specificity for *mcr-1* detection were 96.2% and 98.6%, respectively. The visual limit of detection for the *mcr-1* gene was 3.93 copies/µL [29]. Additionally, validation results with *E. coli* isolates from pig fecal and clinical samples were consistent with PCR-AGE. No cross-reaction was observed with other *mcr* genes and other 7 pathogenic bacteria. Due to the presence of other *mcr* genes, certain strains can exhibit colistin resistance despite lacking the *mcr-1* gene. Therefore, the inconsistent results of RPA-in-house LFA against the phenotypic BMD method were revealed which might be due to chromosomal mutations such as *pmr*AB, *phopQ* genes, other *mcr* variants, or other resistant mechanisms [4]. As a result, the RPA-LFA strategy provides rapid surveillance in field or POC settings to ensure effective therapy and drug resistance control. However, our RPA-LFA specifically detects only the *mcr-1* gene which limits all *mcr* genes screening for detection of colistin-resistant bacteria.

## 4. Materials and Methods

### 4.1. Bacterial Strains for Detection of Colistin Resistance by Phenotyping, PCR and RPA-LFA

A total of 152 *E. coli* isolates were derived from pig fecal samples in our previous study. About 30 fecal samples were randomly collected from healthy pigs in five antibiotic-utilizing farms (D1 = 33, PIG = 28, A1 = 31, A2 = 30, and A3 = 30). Sixty-two clinical isolates of colistin-resistant *E. coli* were obtained from Dr. Pitak Santanirand, Faculty of Medicine, Ramathibodi Hospital, Mahidol University, Bangkok, Thailand, and Dr. Anusak Kerdsin, Faculty of Public Health, Kasetsart University, Chalermphrakiat Sakon Nakhon Province Campus, Thailand, were included in this study to validate the developed RPA-LFA technique. The sample size used in this study was calculated using the Buderer method [30], which helps evaluate the sensitivity and specificity of diagnostic tests at a 95% confidence interval. All bacterial strains were cultured on MacConkey agar and preserved in 20% glycerol at −80 °C for long-term storage.

### 4.2. Phenotypic Colistin Resistance Screening

All 152 *E. coli* isolates obtained from pig fecal samples underwent colistin agar testing following CLSI guidelines (CLSI, 2021). In brief, *E. coli* strains (0.5 McFarland) were diluted at a ratio of 1:10 and then streaked with 10 µL onto the surface of colistin agar plates, ranging from 1 µg/mL to 4 µg/mL. The results were interpreted after incubation at 37 °C for 16 to 20 h. The minimum inhibitory concentration (MIC) was determined to be the lowest concentration on the colistin agar plate that completely inhibited growth, and MIC ≥ 4 µg/mL was reported as resistant.

Following the joint CLSI-EUCAST guideline (EUCAST, 2016), the broth microdilution method was used with colistin-resistant *E. coli* isolates to find reference MICs ranging from 0.125 to 64 µg/mL. Pure colistin sulfate salt powder (Sigma-Aldrich, St. Louis, MO, USA) was used to generate MIC panels of two-fold dilutions in cation-adjusted Mueller Hilton Broth (MHB). Each *E. coli* culture was adjusted to 0.5 McFarland and then further diluted (1:75) with MHB. Twenty-five microliters of the diluted inoculum were added to colistin MIC panels in 96-well plates. MHB alone and inoculum alone served as negative and positive (growth) controls, respectively. The plates were incubated at 37 °C for 16 to 20 h, and the results were visually interpreted for turbidity to determine the MIC. All isolates were tested in duplicate, and quality control strains, including *E. coli* (ATCC 25922) and *Pseudomonas aeruginosa* (ATCC 27853), were used.

### 4.3. DNA Extraction

All the isolates were subjected to DNA extraction by boiling method. Briefly, two to three colonies of each isolate were resuspended in 250 µL of TE buffer, vortexed, incubated at 95 °C for 10 min, and then centrifuged at 13,000 rpm for 5 min. The supernatant was aspirated, and the pellet was resuspended in 200 µL of TE buffer. DNA concentration was quantified at a 260/280 ratio using a microplate reader (BioTek, Winooski, VT, USA). Extracted DNA was stored at −20 °C until further amplification.

### 4.4. PCR for MCR-1 Gene Detection

The PCR method was employed to detect the *E. coli* harboring the *mcr-1* gene in both pig and clinical isolates, incorporating the *usp*A gene as an internal control. Specific primers for the *mcr*-*1* gene, as previously described [31], were used. The PCR was performed in a total of 25 µL, comprising of 1× Standard Taq reaction buffer, 1.5 mM MgCl_2_, 0.2 μM *mcr-1* primers, 0.1 μM *uspA* primers, 0.2 mM dNTPs, 25 ng of DNA template, and 0.625 U of Taq polymerase (New England Biolabs, Wiltshire, UK) The amplification steps were conducted using a thermocycler (Bio-Rad, Hercules, CA, USA) with the following conditions: 94 °C for 5 min, followed by 25 cycles at 94 °C for 1 min, 58 °C for 1 min, 72 °C for 1 min, and post-extension at 72 °C for 5 min. The PCR products were analyzed by 2% agarose gel electrophoresis (AGE). The *E. coli* clone harboring the *mcr-1* gene kindly provided by Dr. Anusak Kerdsin, Faculty of Public Health, Kasetsart University, Chalermphrakiat Sakon Nakhon Province Campus, Thailand, was incorporated as the positive control. The Milli Q water was used as the negative control.

### 4.5. In-House LFA Optimization

The LFA was composed of a sample pad, conjugate pad (soaked with AuNP conjugated anti-biotin), test line/spot (coated with anti-FAM), and control line/spot (coated with goat anti-mouse IgG) imprinted on the membrane, absorbent pad, and backing card (Figure 1). The concentrations of anti-biotin conjugated AuNP (OD2-OD10), goat anti-mouse IgG (0.2–2.4 mg/mL), anti-FITC (0.2–1 mg/mL), and % tween-20 (0.05–1%) in Tris-based buffer were optimized. The positive and negative RPA products were diluted in running buffer (1:10) and 100 μL was applied to the sample pad of each condition. The red color at test and control spots/lines was observed within 5–15 min, and the intensity was measured by Image J software (version 1.53e).

### 4.6. RPA-LFA Optimization

Once the handmade LFA preparation reached its optimal condition, the BioDot ZX1010 dispensing system (Irvine, CA, USA) was set up to spray all antibodies for RPA-LFA optimization. The *mcr*-*1*-specific primer sequence, as described previously [31], was modified with Biotin at the 5′ end and FAM at the 3′ end. The RPA reaction was performed using the TwistAmp Basic reaction kit (TwistDx™, Cambridge, UK). The RPA master mix comprised 14.75 µL of rehydration buffer, 0.3 µM of each forward and reverse primer, and 6.5 µL of Milli Q water. Subsequently, 1 µL (25 ng) of each DNA template, positive DNA control, and Milli Q water were added to each reaction tube, followed by 1.25 µL of 280 mM MgOAc to achieve a total volume of 25 µL. The reaction was incubated at 37 °C for 20 min. To optimize the *mcr*-*1-*RPA reactions, the following conditions were explored: (a) *mcr*-*1* primer concentration—0.48, 0.4, 0.35, and 0.3 µM; (b) temperatures—35 °C, 37 °C, 39 °C, 41 °C, and 43 °C; and (c) incubation times—5, 10, 15, 20, 25, and 30 min. The RPA products were mixed with running buffer and applied to the sample pad of our in-house LFA.

Different ratios of RPA product to running buffer (1:2, 1:4, 1:8, 1:10, 1:16, and 1:20) were tested, and detection times (at 5, 10, 15, 20, 30, and 60 min) were examined to determine the shortest time required to produce a strong red line for both test and control lines. Two visible red lines on the LFA corresponded to the test and control lines, indicating the presence of the *mcr-1* gene. If only one red line appeared on the control line, a test was considered negative. An invalid result was interpreted if no red line appeared on the strips at all, or no red line appeared on the control lines. All LFA results were compared to the results obtained from 2% AGE.

### 4.7. Limit of Detection (LOD) and Specificity of RPA-LFA

Various concentrations of *mcr*-*1*-positive DNA, including 200, 100, 50, 25, 10, 5, 2.5, 1, 0.1, 0.01, and 0.001 ng/L, were used. The LOD was defined as the lowest DNA concentration in the RPA required for amplicon generation. Visualization was done through a naked eye examination using our in-house LFA, and confirmation was performed through AGE.

To assess the specificity of our developed LFA, DNA was isolated from 7 bacterial strains found in clinical and animal specimens, including *E. coli* ATCC 25922, *Proteus mirabilis* ATCC 25933, *Acinetobacter baumannii* ATCC 19606, *K. pneumoniae* ATCC 700603, *Pseudomonas aeruginosa* ATCC 27853, *Enterococcus faecalis* ATCC 29212, and *Staphylococcus aureus* ATCC 25923. In addition, cross-reactivity was investigated using DNA derived from *E. coli* clones containing all *mcr* variants (*mcr-1* to *mcr-10*), which was kindly provided by Dr. Anusak Kerdsin, Faculty of Public Health, Kasetsart University, Chalermphrakiat Sakon Nakhon Province Campus, Thailand.

### 4.8. Validation of the RPA-LFA

A total of 123 *E. coli* isolates, comprising 61 pigs and 62 clinical isolates, were screened for the presence of the *mcr-1* gene using our developed in-house RPA-LFA. The TwistDx basic kit and our in-house LFA were employed under the conditions determined in this study. The obtained results were compared with phenotypic broth microdilution and PCR-AGE. The sensitivity and specificity of the RPA-LFA were analyzed using Medcalc^®^ software (https://www.medcalc.org/calc/diagnostic_test.php) accessed on 8 August 2023.

## 5. Conclusions

In terms of effective AMR control, the last resort antibiotic-resistant genes are seriously alarming. Our RPA-in-house LFA method provides a low-cost, simple, rapid, and portable way to detect *mcr-1* without the need for expensive equipment. Moreover, its high sensitivity, specificity, and accuracy make it a valuable tool for future applications of the *mcr-1* gene monitoring in livestock, clinical settings, and environmental samples. To overcome the limitation in this study, the multiplex RPA-LFA will be further established to screen all *mcr* variants.

## Figures and Tables

**Table 1:** Validation of RPA-LFA with broth microdilution, and PCR.

	Phenotypic BMD		PCR	
RPA-LFA	Colistin resistance	Colistin susceptible	MCR1+	MCR1−
MCR1+	65	0	65	0
MCR1−	25	33	0	58
Total	90	33	65	58
	Sensitivity 72.2% CI 61.78% to 81.15%		Sensitivity 100.00% CI 94.48% to 100.00%	
	Specificity 100.00% CI 89.42% to 100.00%		Specificity 100.00% CI 93.84% to 100.00%	
	PPV 100.00% CI 94.48% to 100.00%		PPV 100.00% CI 94.48% to 100.00%	
	NPV 56.9% CI 48.61% to 64.81%		NPV 100.00% CI 93.84% to 100.00%	
	Accuracy 76.67% CI 71.48% to 86.39%		Accuracy 100.00% CI 97.0522% to 100.00%	

## Data Availability

Data is contained within the article. The original contributions presented in the study are included in the article, further inquiries can be directed to the corresponding author/s.

## References

[1] El-Sayed Ahmed M.A.E.-G., Zhong L.-L., Shen C., Yang Y., Doi Y., Tian G.-B. (2020). Colistin and its role in the era of antibiotic resistance: An extended review (2000–2019). Emerg. Microbes Infect..

[2] Mondal A.H., Khare K., Saxena P., Debnath P., Mukhopadhyay K., Yadav D. (2024). A Review on colistin resistance: An antibiotic of last resort. Microorganisms.

[3] Rhouma M., Madec J.-Y., Laxminarayan R. (2023). Colistin: From the shadows to a one health approach for addressing antimicrobial resistance. Int. J. Antimicrob. Agents.

[4] Velkov T., Roberts K.D., Nation R.L., Thompson P.E., Li J. (2013). Pharmacology of polymyxins: New insights into an ‘old’class of antibiotics. Future Microbiol..

[5] Anyanwu M.U., Jaja I.F., Okpala C.O.R., Njoga E.O., Okafor N.A., Oguttu J.W. (2023). Mobile colistin resistance (*mcr*) gene-containing organisms in poultry sector in low-and middle-income countries: Epidemiology, characteristics, and one health control strategies. Antibiotics.

[6] Hussein N.H., Al-Kadmy I.M.S., Taha B.M., Hussein J.D. (2021). Mobilized colistin resistance (*mcr*) genes from 1 to 10: A comprehensive review. Mol. Biol. Rep..

[7] Lobato I.M., O’Sullivan C.K. (2018). Recombinase polymerase amplification: Basics, applications and recent advances. Trac Trends Anal. Chem..

[8] (2021). Performance Standards for Antimicrobial Susceptibility Testing. Thirty-One Informational Supplement.

[9] Tan T.Y., Ng S.Y. (2007). Comparison of Etest, Vitek and agar dilution for susceptibility testing of colistin. Clin. Microbiol. Infect..

[10] European Committee on Antimicrobial Susceptibility Testing (2016). Recommendations for MIC Determination of Colistin (Polymyxin E) as Recommended by the Joint CLSI-EUCAST Polymyxin Breakpoints Working Group.

[11] Daniels J.B., Campbell D., Boyd S., Ansari U., Lutgring J., Rasheed J.K., Halpin A.L., Sjölund-Karlsson M. (2019). Development and validation of a clinical laboratory improvement amendments-compliant multiplex real-time PCR assay for detection of *mcr* genes. Microb. Drug Resist..

[12] Zou D., Huang S., Lei H., Yang Z., Su Y., He X., Zhao Q., Wang Y., Liu W., Huang L. (2017). Sensitive and rapid detection of the plasmid-encoded colistin-resistance gene *mcr-1* in *Enterobacteriaceae* isolates by loop-mediated isothermal amplification. Front. Microbiol..

[13] Wang Y., Chen H., Pan Q., Wang J., Jiao X., Zhang Y. (2024). Development and evaluation of rapid and accurate one-tube RPA-CRISPR-Cas12b-based detection of *mcr-1* and *tet (x4)*. Appl. Microbiol. Biotechnol..

[14] Bahadır E.B., Sezgintürk M.K. (2016). Lateral flow assays: Principles, designs and labels. TrAC Trends Anal. Chem..

[15] Koczula K.M., Gallotta A. (2016). Lateral flow assays. Essays Biochem..

[16] Zheng T., Li X., Si Y., Wang M., Zhou Y., Yang Y., Liang N., Ying B., Wu P. (2023). Specific lateral flow detection of isothermal nucleic acid amplicons for accurate point-of-care testing. Biosens. Bioelectron..

[17] Liu J., Zhang Z., Feng Y., Hu H., Yu Y., Qiu L., Liu H., Guo Z., Huang J., Du C. (2020). Molecular detection of the *mcr* genes by multiplex PCR. Infect. Drug Resist..

[18] Lu C., Wang J., Pan L., Gu X., Lu W., Chen D., Zhang C., Ye Q., Xiao C., Liu P. (2023). Rapid detection of multiple resistance genes to last-resort antibiotics in *Enterobacteriaceae* pathogens by recombinase polymerase amplification combined with lateral flow dipstick. Front. Microbiol..

[19] Kanokudom S., Assawakongkarat T., Akeda Y., Ratthawongjirakul P., Chuanchuen R., Chaichanawongsaroj N. (2021). Rapid detection of extended spectrum β-lactamase producing *Escherichia coli* isolated from fresh pork meat and pig cecum samples using multiplex recombinase polymerase amplification and lateral flow strip analysis. PLoS ONE.

[20] Khine N.O., Lugsomya K., Niyomtham W., Pongpan T., Hampson D.J., Prapasarakul N. (2022). Longitudinal monitoring reveals persistence of colistin-resistant *Escherichia coli* on a pig farm following cessation of colistin use. Front. Vet. Sci..

[21] Wang Y., Xu C., Zhang R., Chen Y., Shen Y., Hu F., Liu D., Lu J., Guo Y., Xia X. (2020). Changes in colistin resistance and *mcr-1* abundance in *Escherichia coli* of animal and human origins following the ban of colistin-positive additives in china: An epidemiological comparative study. Lancet Infect. Dis..

[22] Peng Z., Zhang X., Li X., Hu Z., Li Z., Jia C., Dai M., Tan C., Chen H., Wang X. (2021). Characteristics of colistin-resistant *Escherichia coli* from pig farms in central China. Anim. Dis..

[23] Shen Y., Zhang R., Schwarz S., Wu C., Shen J., Walsh T.R., Wang Y. (2020). Farm animals and aquaculture: Significant reservoirs of mobile colistin resistance genes. Environ. Microbiol..

[24] Bastidas-Caldes C., de Waard J.H., Salgado M.S., Villacís M.J., Coral-Almeida M., Yamamoto Y., Calvopiña M. (2022). Worldwide prevalence of mcr-mediated colistin-resistance *Escherichia coli* in isolates of clinical samples, healthy humans, and livestock—A systematic review and meta-analysis. Pathogens.

[25] Zhong L.-L., Zhou Q., Tan C.-Y., Roberts A.P., El-Sayed Ahmed M.A.E.-G., Chen G., Dai M., Yang F., Xia Y., Liao K. (2019). Multiplex loop-mediated isothermal amplification (multi-lamp) assay for rapid detection of *mcr-1* to *mcr-5* in colistin-resistant bacteria. Infect. Drug Resist..

[26] Xu J., Wang X., Yang L., Kan B., Lu X. (2018). Rapid detection of *mcr-1* by recombinase polymerase amplification. J. Med. Microbiol..

[27] Li J., Macdonald J., von Stetten F. (2020). Correction: Review: A comprehensive summary of a decade development of the recombinase polymerase amplification. Analyst.

[28] Gong L., Jin Z., Liu E., Tang F., Yuan F., Liang J., Wang Y., Liu X., Wang Y. (2022). Highly sensitive and specific detection of mobilized colistin resistance gene *mcr-1* by CRISPR-based platform. Microbiol. Spectr..

[29] Tao J., Liu D., Xiong J., Dou L., Zhai W., Zhang R., Wang Y., Shen J., Wen K. (2022). Rapid on-site detection of extensively drug-resistant genes in enterobacteriaceae via enhanced recombinase polymerase amplification and lateral flow biosensor. Microbiol. Spectr..

[30] Buderer N.M.F. (1996). Statistical Methodology: I. Incorporating the prevalence of disease into the sample size calculation for sensitivity and specificity. Acad. Emerg. Med..

[31] Rebelo A.R., Bortolaia V., Kjeldgaard J.S., Pedersen S.K., Leekitcharoenphon P., Hansen I.M., Guerra B., Malorny B., Borowiak M., Hammerl J.A. (2018). Multiplex PCR for detection of plasmid-mediated colistin resistance determinants, *mcr-1*, *mcr-2*, *mcr-3*, *mcr-4* and *mcr-5* for surveillance purposes. Eurosurveillance.

